# Single-cell analysis of peripheral blood and pleural effusion reveals functional diversity of γδ T cells in tuberculosis infection

**DOI:** 10.3389/fimmu.2025.1605827

**Published:** 2025-06-18

**Authors:** Yanjun Feng, Yu Chen, Wanying Zhang, Xinghua Shen, Jinyu Yan, Lin Yao, Lijuan Zhang, Yayan Niu, Jianping Zhang, Peijun Tang, Chunhua Ling

**Affiliations:** ^1^ Department of Pulmonary and Critical Care Medicine, The First Affiliated Hospital/Department of Tuberculosis, The Fifth People’s Hospital of Suzhou & The Affiliated Infectious Diseases Hospital, Suzhou Medical College of Soochow University, Suzhou, China; ^2^ Department of Biochemistry and Molecular Biology, School of Biology and Basic Medical Sciences, Suzhou Medical College of Soochow University, Suzhou, China

**Keywords:** tuberculosis, Mycobacterium tuberculosis, γδ T cells, FCGR3a, single-cell analysis

## Abstract

**Introduction:**

Tuberculosis is a contagious airborne disease caused by the Mycobacterium tuberculosis infection. γδ T cells are closely associated with TB infection; however, the specific role of γδ T cells in the immune response to TB remains unclear, as does the differentiation and mechanism of γδ T cell subsets in TB patients.

**Methods:**

We analyzed the characteristics of γδ T subsets in the peripheral blood (Peripheral Blood Mononuclear Cells,PBMC) and pleural effusions (Tuberculous pleural effusion,TPE) and pleural effusions of TB patients using single-cell sequencing to explore the distribution and characteristics of different γδ T subpopulations.

**Results:**

Seven γδ Tcell subpopulations were identified. The highest percentage of effector γδ2 cell cluster (C1) was found in PBMCs from TPE patients, accounting for 36.1%, while the highest percentage of tissue-resident γδ2 cell cluster (C0) was found in PFMCs, reaching 70.5%. Through in-depth analysis, we identified a group of Vδ2 cells exhibiting strong effector function and high expression of *FCGR3A*.

**Discussion:**

Therefore, exploring the mechanism of interaction between Vδ2 cells and Mtb, as well as understanding host immune regulation during Mtb infection, can not only enhance the understanding of the immune mechanism underlying TB but also provide new theoretical ideas. This research may offer novel therapeutic targets for TB and innovative strategies for treatment and prevention.

## 1 Introduction

Tuberculosis (TB), a disease caused by the Mycobacterium tuberculosis complex (MTBC), has become one of the world’s deadliest infectious diseases and poses a serious challenge to global health (1). The absence of innovative diagnostic tools, novel therapeutics and effective vaccines presents a major challenge for TB prevention and control, underscoring the importance of research into effective treatment modalities. The development of TB vaccines and immunotherapies represents a promising strategy to combat this disease. γδ T cells are a unique subsets of T cells with intrinsic immune functions, characterized by their T cell receptor (TCR), which consists of γ and δ chains. Based on the expression of the δ chain of the TCR on the surface of γδ T cells, these cells can be classified into three subpopulations: Vδ1 T cells, Vδ2 T cells (also known as Vγ9Vδ2 T cells), and Vδ3 T cells (2). Vδ2 T cells are predominantly found in the peripheral blood, accounting for 50% to 90% of the total γδ T cell population (3). The TCR γδ on the surface of Vδ2 T cells mainly adopts the pairing of Vγ9 and Vδ2, and is capable of recognizing and activating phosphorylated antigens and secreting perforin, granzyme, etc. to produce cytotoxicity (4). Activated Vδ2 T cells can function as antigen-presenting cells (5). Vδ2 T cells also play an important role in TB (6). They bind to the TCR-CD3 complex via the Mycobacterium tuberculosis phosphoantigen isopentenyl pyrophosphate (IPP), which promotes the production of TNF-α and exerts an anti-tuberculosis effect (7, 8). We conducted single-cell sequencing on peripheral blood and pleural effusion samples from five patients with tuberculous pleurisy (TPE) (9) and analyzed the γδ T cell subset profiles of TPE patients to identify novel effector Vδ2 T-cell potential marker genes, which may provide new therapeutic targets and strategies for the treatment and prevention of TB.

## 2 Methods

### 2.1 Single cell sequencing data download and processing

The single-cell datasets HRA000910 and HRA000363, which contain 13 samples, were downloaded from the National Genomics Data Centre (NGDC) Genome Sequence Archive (GSA). The raw data from each sample were subjected to sequencing library demultiplexed and aligned to the human reference genome GRCh38, followed by quantification of UMI counts using 10x Genomics Cell Ranger (v3.1.0). After cell identification using DropletUtils (v1.6.1), the quantified expression matrix was QC’d using the Seurat package (v5.1.0) for R software (v4.4.1). Cells with less than <15% mitochondrial gene content, together with a total gene count >300 and gene expression level between 500 and 15,000 and expressed in at least three cells were retained.

### 2.2 γδ T cell filtering and dimensionality reduction clustering

After quality control, the data were filtered for γδ T cells based on PTPRC, CD3D, CD3E, TRDV1, and TRDV2 gene expression levels greater than 1 using the subset function. The gene expression data were normalized using the NormalizeData function, which employs the default parameters of the Seurat package, the 2000 genes with the largest variations were selected using the FindVariableFeatures function, the gene expression was normalized using the ScaleData function and finally the data were linearly downscaled by PCA using the RunPCA function. The FindNeighbors function was performed by selecting 1:15 PCs based on the ElbowPlot function, and the FindClusters function was performed with a resolution of 0.6. For visual clustering, the RunUMAP function was used with the same number of PCs to generate the Uniform Manifold Approximation and Projection (UMAP) algorithm.

### 2.3 Cluster cell type identification

Marker genes for each cluster relative to all other clusters were determined using the FindAllMarkers, employing a Wilcoxon test with a p-value <0.05, Bonferroni correction test with p-adj < 0.05 and a differential expression threshold of 0.25, where selected marker genes were expressed in at least 25% of the target cell subpopulations. Each cluster was determined to specifically express the top 3 genes using the COSG package (v0.9.0). The method for gene marker identification based on cosine-based values. Cluster was labeled by manually matching typical cell marker genes with the algorithmically calculated genes that are characteristic of each cluster.

### 2.4 Analysis of cell cycle

G1/S and G2/M phase signature genes were analyzed using the CellCycleScoring function to predict the temporal cell cycle phases of γδ T cell subsets.

### 2.5 Pseudotime analysis of scRNA-seq

Cell trajectories were analyzed using the monocle package (v2.32.0) for the Vδ1 and Vδ2 cell clusters, respectively. Seurat data were analyzed downstream for differential gene expression using a negative binomial distribution model with the newCellDataSet. Normalization was performed using the estimateSizeFactors and estimateDispersions functions, and feature selection was performed with the differentialGeneTest function to filter out 2000 genes that had a significant impact on cell trajectories. The reduceDimension function was employed to reduce the dimensionality of the data using the DDRTree algorithm, and the orderCells function was used to sort the cells according to their developmental trajectory. The BEAM function was used to model and analyze the branch point-dependent genes within the cell developmental trajectory to obtain the significance score for each gene.

### 2.6 Pathway enrichment analysis

Enrichment analyses were performed for each cell cluster using the clusterProfiler package (v4.12.6) and the compareCluster function to select the enrichGO Biological Processes (BP) GO database. P-values were corrected using the Benjamini & Hochberg (BH) method. Additionally, pathway enrichment analysis was performed for each cell cluster using the ReactomePA package (v1.48.0) with the compareCluster function to select the Reactome database.

## 3 Results

### 3.1 Patients with TPE single-cell sequencing results in PFMCs are distinct from PBMCs

**Figure 1:**
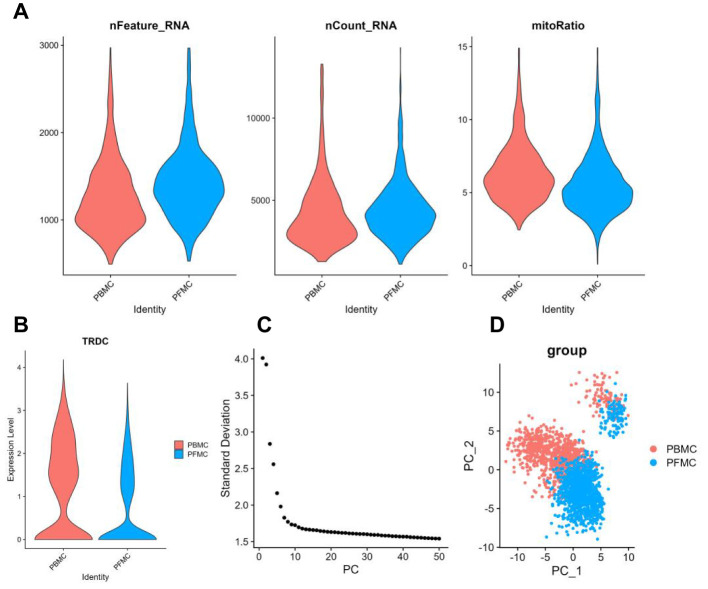
Single-cell analysis of PBMCs and PFMCs from TPE patients **(A)** Violin plots of the number of genes, number of counts and percentage of mitochondrial genes accounted for after quality control of single-cell data in PBMCs and PFMCs from TPE patients. **(B)** Violin plots of *TRDV* gene expression levels in PBMCs and PFMCs from TPE patients. **(C)** Principal component fragmentation plots of γδ T cell single cell data. **(D)** Visualisation of the principal component analysis of single cell data from PBMCs and PFMCs of TPE patients.

We obtained fresh mononuclear cells from peripheral blood (PBMC) and pleural fluid (PFMC) of five TPE patients for single-cell sequencing (9), and used the Seurat R package (10) for quality control and screening of the raw sequencing data for γδ T cells (*TRDC*) (**Figures 1A, B**). Ultimately, 959 γδ T cells from peripheral blood and 1259 γδ T cells from pleural fluid were included in the study. The screened γδ T cells were linearly downscaled using principal component analysis (PCA), and the top 15 principal components were then selected for downscaled clustering based on the fragmentation map (**Figures 1C, D**). The results of the PCA demonstrated that there is a distinction in the transcriptomic profiles exhibited by PBMCs and Pleural Fluid Mononuclear Cells (PFMCs) in TPE patients.

### 3.2 Single-cell transcriptomics reveals γδ T cell atlas between PBMCs and PFMCs from patients with TPE

**Figure 2:**
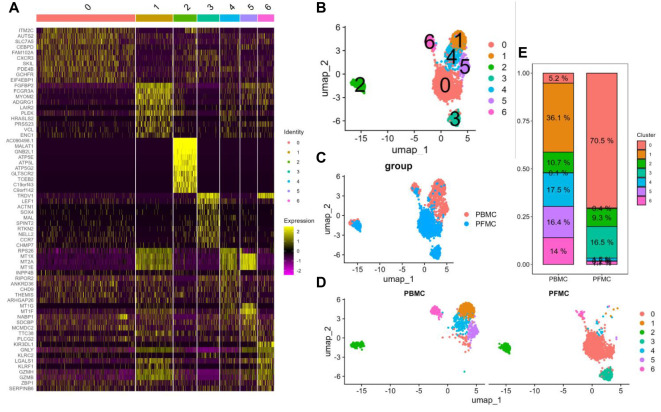
Single-cell transcriptional landscape of γδ T cells in PBMCs and PFMCs from TPE patients **(A)** Heatmap of the top 10 highly variable genes in each cluster of γδ T cells. **(B)** UMAP visualisation of PBMCs and PFMCs single cell data from TPE patients. **(C)** UMAP visualisation of each γδ T cell cluster. **(D)** UMAP visualisation of each γδ T cell cluster in PBMCs and PFMCs. **(E)** Percentage of each γδ T cell cluster in PBMCs and PFMCs from TPE patients.

The unsupervised clustering algorithm yielded a total of 7 γδ T cell subpopulations ranging from 0 to 6, and the top 10 gene heatmaps for each cluster were as follows (**Figure 2A**). Using the non-linear clustering UMAP method (11) to visualise the γδ T cell subpopulations, we classified γδ T cells into 7 clusters (**Figure 2B**). Consistent with previous PCA results, UMAP visualization results showed that PBMCs and PFMCs from TPE patients have distinct clusters (**Figure 2C**). Clusters C1, C4, C5, and C6 had a higher proportion of PBMCs with 36.1%, 17.5%, 16.4%, and 14%, respectively, while PFMCs were more predominant in clusters C0 and C3 with 70.5% and 16.5%, respectively, and cluster C2 is the same in both (**Figures 2D, E**).

### 3.3 Heterogeneity of γδ T cell subsets

**Figure 3:**
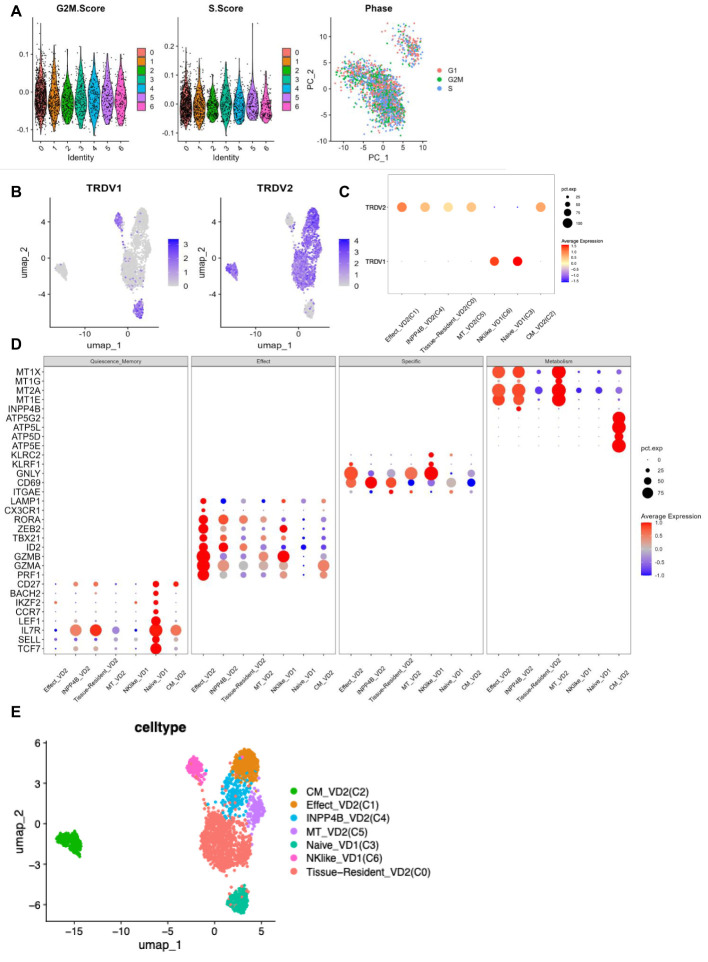
γδ T cell clusters annotation **(A)** G2M phase scoring violin plot for each cluster of γδ T cells (left panel) S phase scoring violin plot (middle panel) Visualisation of cell cycle principal component analysis (right panel). **(B)** *TRDV1* expression in each cluster of γδ T cells (left panel) *TRDV2* expression in each cluster of γδ T cells (right panel). **(C)** *TRDV1* and *TRDV2* expression dot plots in each cluster of γδ T cells. **(D)** Expression dot plots of some signature molecules in different clusters of γδ T cells. **(E)** UMAP visualisation of γδ T cell clusters after annotation.

According to the expression of γδ T cell signature molecules (*TRDV1*, *TRDV2*), C3 and C6 were identified as Vδ1 cells, while C0, C1, C2, C4, and C5 were classified as Vδ2 cells (**Figure 3A**). The scoring calculation of the cell cycle status (12) for each γδ T cell cluster showed that all seven γδ T cell subpopulations exhibited the same cell cycle status (**Figures 3B, C**).

The highest percentage of effector Vδ2 cell cluster (C1) (36.1%), along with elevated expression of cytotoxicity-related genes (*PRF1*, *GZMA*, *GZMB*, *CX3CR1*, and *LAMP1*) and effector transcription factors (*ID2*, *TBX21*, and *ZEB2*) were found in PBMCs from TPE patients. The highest percentage (70.5%) of tissue-resident Vδ2 cell cluster (C0) was found in PFMCs, with high expression of tissue-resident related genes (*ITGAE* and *CD69*) *(* 13), and its high expression of IL7R was speculated to be a possible tissue-resident memory Vδ2 cell subpopulation. Central memory Vδ2 cells (C2) was found in both PBMCs and PFMCs, expressing high levels of CD27, IL7R and mitochondrial oxidative phosphorylation genes (*ATP5E*, *ATP5D*, etc.) (14), as well as cytotoxicity-related genes (*PRF1*, *GZMA*). In PFMCs, there was a higher percentage (16.5%) of naive Vδ1 cell cluster (C3), characterized by elevated expression of resting stemness-related genes (*TCF7*, *SELL*, *LEF1*, *BACH2*, and *IKZF2*) *(* 15) and almost no expression of effector-related genes. In PBMC, the INPP4B^+^ Vδ2 cell cluster (C4) accounted for 17.5% and was characterized by high expression of the inositol phosphatase INPP4B, along with elevated expression of metallothionein-related genes (*MT1E* and *MT2A*) (16) (**Figures 3D, E**). The MT^+^ Vδ2 cell cluster (C5) accounted for (16.4%) in PBMCs, which was characterized by high expression of metallothionein-related genes (*MT1E*, *MT2A*, *MT1G*, and *MT1X*) *(* 17, 18). The NK-like Vδ1 cell cluster (C6) accounted for (14%) in PBMCs, which was characterized by high expression of NK-related genes (*GNLY*, *KLRC2*, and *KLRF1*) *(* 19, 20), as well as high expression of cytotoxicity-associated genes (*PRF1*, *GZMA*, *GZMB*, *CX3CR1*, and *LAMP1*) and effector transcription factors (*TBX21* and *ZEB2*) (**Figures 3D, E**). Taken together, these results reflect the phenotypic and functional heterogeneity of γδ T cells in PBMCs and PFMCs of TPE patients.

### 3.4 Trajectory analysis of Vδ2 and Vδ1 T cell subsets among PBMCs and PFMCs

**Figure 4:**
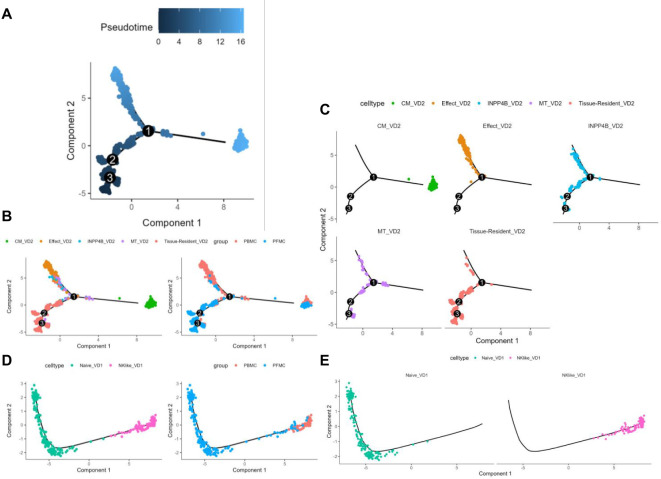
γδT cell subsets pseudotime analysis **(A)** Colour visualisation of Vδ2 cell clusters according to pseudotime. **(B)** Cell visualisation according to the Vδ2 cell cluster for the pseudotime arrangement (left panel) Cell visualisation according to the pseudotime arrangement of PBMCs and PFMCs groupings in TPE patients (right panel). **(C)** Annotated faceted display according to Vδ2 cell clusters. **(D)** Pseudotime arrangement of cell visualisation according to Vδ1 cell cluster (left panel) Pseudotime arrangement of cell visualisation according to PBMCs and PFMCs grouping in TPE patients (right panel). **(E)** Annotated faceted display according to Vδ1 cell clusters.

We analyzed the differentiation trajectories of Vδ2 cell subpopulations using pseduotime analysis (21) and found that the tissue-resident Vδ2 cell cluster (C0) was predominantly located in the lower left branch, whereas the effector Vδ2 cell cluster (C1) and the central memory Vδ2 cell cluster (C2) were located in the upper left and right branches, respectively (**Figures 4A–C**). Based on pseduotime calculations, it was speculated that the tissue-resident Vδ2 cell cluster (C0) mainly differentiated into the effector Vδ2 cell cluster (C1) and the central memory Vδ2 cell cluster (C2), whereas the INPP4B^+^ Vδ2 cell cluster (C4) and the MT^+^ Vδ2 cell cluster (C5) were mainly located in the intermediate transition state (**Figures 4A–C**). In contrast, the proposed time series analysis of the differentiation trajectory of the Vδ1 cell cluster showed that there was no branching of the differentiation trajectory, and the naive Vδ1 cell cluster (C3) differentiated into the NK-like Vδ1 cell cluster (C6) (**Figures 4D, E**). We speculate that the Vδ2 cluster *in situ* in the lungs of TPE patients stimulated by TPE infection has two main directions of differentiation, one into the peripheral blood to differentiate into a Vδ2 cluster with effector functions, and the other to become a central memory Vδ2 cluster to cope with TPE in the long term. Overall, these results reveal the differentiation trajectory of γδ T cell subsets.

### 3.5 Characterizing FCGR3A^+^ Vδ2 cells as a novel subset

**Figure 5:**
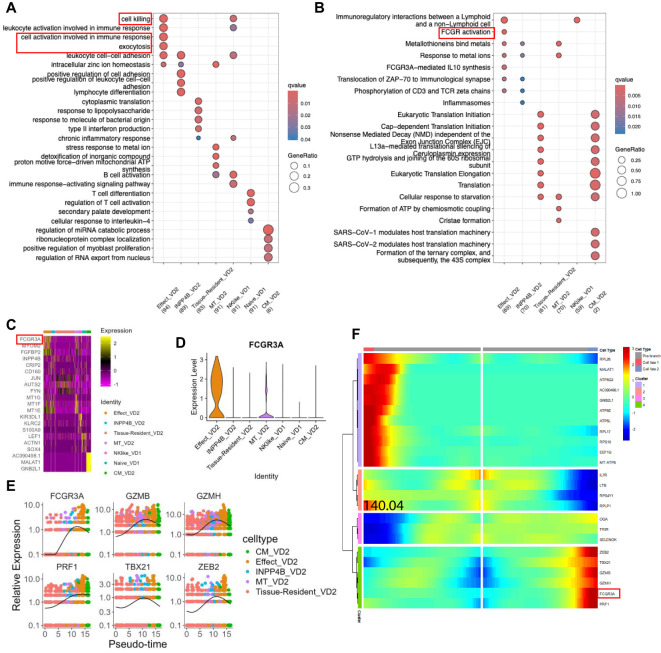
Effector Vδ2 cell subset specific expression of FCGR3A **(A)** GO database bioprocess enrichment analysis dot plot. **(B)** Dot plot of pathway enrichment analysis from the Reactome database. **(C)** Heatmap of the top 3 featured genes in each subpopulation of γδ T cells screened by the COSG package. **(D)** Violin plot of FCGR3A expression in each cluster of γδ T cells. **(E)** Expression changes of some genes in different cluster of Vδ2 cells over time. **(F)** Heatmap of branch-dependent related gene expression screened by the BEAM algorithm.

Effector Vδ2 cells are the mainstay of the TPE immune response, and further study of the functions and characteristic genes of these effector Vδ2 cells can enhance our understanding of the TPE immune response of γδ T cells. Enrichment analysis of biological processes in the GO database22 revealed that a cluster of effector Vδ2 cells exhibated high activation and cytotoxic functions (**Figure 5A**; **Supplementary Material 3**). Enrichment analysis of the Reactome database23 revealed that a cluster of effector Vδ2 cells had specific FCGR-activated signaling pathways (**Figure 5B**; **Supplementary Material 3**). We further analyzed the characteristic genes of each cluster using the COSG package24, in which the characteristic gene of the effector Vδ2 cell subpopulation was FCGR3A, and the violin plots also showed that FCGR3A was highly expressed in the effector Vδ2 cell subset (**Figures 5C, D**). The expression of FCGR3A increased with the differentiation of Vδ2 cells and reached the highest level in the effector Vδ2 cell subset, and at the same time, the expression of FCGR3A with the other cytotoxicity-related genes (PRF1, GZMB and GZMH) and effector transcription factors (TBX21 and ZEB2) had similar pseudotime expression patterns (**Figure 5E**). We used a regression algorithm to search for pseudotime differential genes in the effector Vδ2 cell cluster, and the heatmap showed FCGR3A as a branching-dependent gene in the effector Vδ2 cell subset (q-value < 0.001) (**Figure 5F**; **Supplementary Material 1**).

In conclusion, CD16 (encoded by the *FCGR3A* gene) was specifically expressed in a subset of effector Vδ2 cells in the peripheral blood of TPE patients and correlated with cytotoxic and effector transcription factor expression.

## 4 Discussion

Activated Vδ2 T cells secrete a variety of cytokines and chemokines (22, 23). In the context of bacterial infections, such as Mtb, the immune system employs a range of cytokines and chemokines. Among these are Th1-type cytokines, including γ-interferon-γ (interferon-γ, IFN-γ), tumor necrosis factor-α (TNF-α), and Th17-type cytokines, such as interleukin-17A (IL-17A) (24–26). These cytokines play a crucial role in immune defense. Furthermore, activated Vδ2 T cells have demonstrated significant cytotoxic activity through the death receptor/death receptor ligand (factor-related apoptosis/factor-related apoptosis ligand, Fas/FasL) and granzyme/perforin pathways (27). Furthermore, an examination of other soluble factors produced by Vδ2 T cells revealed a significant correlation between the level of GZMA production and the inhibition of intracellular Mtb growth (27). GZMA proteins secreted by activated Vδ2 T cells are internalised within infected cells, ultimately inhibiting intracellular Mycobacterium growth, lysing infected macrophages, and limiting the spread of bacterial diffusion. Consequently, understanding of the mechanisms of Vδ2 T cell-Mtb interactions and the host immune regulation during Mtb infection contributes to the development of anti-tuberculosis therapies.

In this study, We re-analyzed the peripheral blood and pleural effusions of TB patients by single cell sequencing and revealed significant phenotypic and functional differences among these subpopulations (9). It was found that Vδ2 T cells predominated in the peripheral blood, especially the effector Vδ2 cell subpopulation (C1), which exhibited high expression of genes associated with cytotoxicity (e.g. *PRF1*, *GZMA*, *GZMB* etc.), and effector transcription factors (e.g., *TBX21*, *ZEB2*), suggesting that these cells posses a potent cell-killing function in the anti-tuberculosis immune response. Conversely, a higher percentage of the tissue-resident Vδ2 cell subpopulation (C0) was identified in pleural effusions, suggesting that these cells may play an important role in the local immune response. Furthermore, the study revealed the naïve state of Vδ1 T cells in pleural effusions (C3) and the NK-like state in peripheral blood (C6), thus demonstrating the functional diversity of γδ T cells in distinct tissue environments.

The present study utilized pseudotime analysis to elucidate the differentiation trajectory of Vδ2 T cells. The tissue-resident Vδ2 cell subpopulation (C0) appears to differentiate into two distinct subpopulations: the effector Vδ2 cell subpopulation (C1) and the central memory Vδ2 cell subpopulation (C2). This finding suggests the possibility of distinct differentiation pathways for these cells in response to varying immune demands following TB infection. The higher percentage of the effector Vδ2 cell subpopulation (C1) in peripheral blood suggests that these cells may play an important role in the systemic immune response, whereas the central memory Vδ2 cell subpopulation (C2) may contribute to the formation of long-term immune memory. In addition, the differentiation trajectory of Vδ1 T cells was relatively simple, with the naïve Vδ1 cell subpopulation (C3) differentiating into the NK-like Vδ1 cell subpopulation (C6), suggesting a single pathway of Vδ1 T cell differentiation following TB infection.

A notable finding of this study was the specific expression of CD16 (encoded by the *FCGR3A* gene) in a subpopulation of effector Vδ2 cells. CD16 is an Fcγ receptor commonly associated with the cytotoxic function of natural killer cells (NK cells). The study revealed that *FCGR3A* was expressed at a significantly higher level in a specific subpopulation of effector Vδ2 cells and exhibited a comparable expression pattern to that of genes associated with cell killing (e.g., *PRF1*, *GZMB*, etc.), and effector transcription factors (e.g., *TBX21*, *ZEB2*). This finding suggests that CD16 may play a significant role in the cell-killing function of effector Vδ2 cells. Furthermore, the expression of *FCGR3A* increased with the differentiation of Vδ2 cells, thereby further supporting the critical role of CD16 in effector Vδ2 cell function. This finding provides a new perspective for understanding the mechanism of γδ T cells in the immune response to TB and may provide potential targets for future immunotherapy.

Despite the findings of the present study, which revealed the functional diversity of γδ T cells in the immune response to tuberculosis, there are several limitations that must be considered. Firstly, the sample size was relatively small, and larger studies are needed to validate these findings in the future. Secondly, the study primarily focused on the function and differentiation trajectory of γδ T cells, while the interactions of these cells with other immune cells (e.g., monocytes, dendritic cells, etc.) remain a subject for future investigation. This exploration will contribute to a more comprehensive understanding of the immune response mechanism of TB. Furthermore, the present study is primarily based on transcriptomic data, which can be combined with proteomics and functional experiments in the future research to further validate the function and mechanism of γδ T cells.

In conclusion, this study revealed the functional diversity and differentiation trajectory of γδ T cells in tuberculosis patients, especially the critical role of Vδ2 T cells in anti-tuberculosis immunity by single-cell RNA sequencing. The study demonstrated that the effector Vδ2 cell subpopulation exhibit high cytotoxic function, with CD16 (*FCGR3A*) being specifically expressed in this subpopulation, suggesting its role in the immune response to TB. These findings provide novel insights into the mechanisms of γδ T cells in TB immunity and suggest potential targets for future immunotherapy.

## Data Availability

Publicly available datasets were analyzed in this study. This data can be found here: The single-cell datasets HRA000910 and HRA000363, which contain 13 samples, were downloaded from the National Genomics Data Centre (NGDC) Genome Sequence Archive (GSA).
